# Community Water Improvement, Household Water Insecurity, and Women’s Psychological Distress: An Intervention and Control Study in Ethiopia

**DOI:** 10.1371/journal.pone.0153432

**Published:** 2016-04-28

**Authors:** E. G. J. Stevenson, A. Ambelu, B. A. Caruso, Y. Tesfaye, M. C. Freeman

**Affiliations:** 1 Department of Anthropology, University College London, London, United Kingdom; 2 Hubert Department of Global Health, Emory University, Atlanta, GA, United States of America; 3 Department of Environmental Health Sciences, Jimma University, Jimma, Ethiopia; 4 Department of Behavioral Science and Health Education, Emory University, Atlanta, GA, United States of America; 5 Department of Anthropology, Oregon State University, Corvallis, OR, United States of America; 6 Department of Environmental Health, Emory University, Atlanta, GA, United States of America; Massachusetts General Hospital, UNITED STATES

## Abstract

**Background:**

Over 650 million people worldwide lack access to safe water supplies, and even among those who have gained access to ‘improved’ sources, water may be seasonally unreliable, far from homes, expensive, and provide insufficient quantity. Measurement of water access at the level of communities and households remains crude, and better measures of household water insecurity are urgently needed to inform needs assessments and monitoring and evaluation. We set out to assess the validity of a quantitative scale of household water insecurity, and to investigate (1) whether improvements to community water supply reduce water insecurity, (2) whether water interventions affect women’s psychological distress, and (3) the impacts of water insecurity on psychological distress, independent of socio-economic status, food security, and harvest quality.

**Methods and Findings:**

Measures were taken before and one to six months after a community water supply improvement in three villages in rural northern Ethiopia. Villages similar in size and access to water sources and other amenities did not receive interventions, and served as controls. Household water insecurity was assessed using a 21-item scale based on prior qualitative work in Ethiopia. Women’s psychological distress was assessed using the WHO Self-Reporting Questionnaire (SRQ-20). Respondents were either female heads of household or wives of the heads of household (n = 247 at baseline, n = 223 at endline); 123 households provided data at both rounds. The intervention was associated with a decline of approximately 2 points on the water insecurity scale between baseline and endline compared to the control (beta -1.99; 95% CI’s -3.15, -0.84). We did not find evidence of impact of the intervention on women’s psychological distress. Water insecurity was, however, predictive of psychological distress (p <0.01), independent of household food security and the quality of the previous year’s harvest.

**Conclusion:**

These results contribute to the construct validity of our water insecurity scale, and establish our approach to measuring water insecurity as a plausible means of evaluating water interventions. Improvements to community water supplies were effective in reducing household water insecurity, but not psychological distress, in this population. Water insecurity was an important predictor of psychological distress. This study contributes to an emerging literature on quantitative assessment of household water insecurity, and draws attention to the potential impact of improved access to water on women’s mental well-being.

## Introduction

Despite progress over past decades in expanding access to clean drinking water, 4 billion people (two-thirds of the world’s population) face severe water scarcity each year [1], and an estimated 663 million people remain without access to water sources protected from outside contamination [2]. The use of microbiologically unsafe drinking water is a substantial contributor to diarrheal disease burden [3], and has been associated with helminth infection [4] and impaired physical growth [5] among young children. Water is deservedly recognized–for example in the Sustainable Development Goals–as a crucial determinant of population health and development.

Despite the widely acknowledged importance of reliable water supply for population health, current measures of water access are crude. The standard indicator of water access, as defined by the WHO/UNICEF Joint Monitoring Programme, is that the household’s primary source for drinking is ‘improved,’ implying protection from microbiological contamination [6]. However, even among those who have access to ‘improved’ sources, water may be seasonally unreliable, far from homes, expensive, and provide insufficient quantity. In search of better measures of water access, recent work has drawn on approaches from studies of food security. By analogy to *food* insecurity, household *water* insecurity is defined as insecure access to adequate water for a healthy lifestyle [7]. By asking household members a short list of questions about their experiences (e.g. skipping meals or eating less desirable foods) researchers have devised scales of food insecurity that have proven valid in many settings [8–10]. By adapting this approach to the measurement of water insecurity, it might be possible quickly to assess household water access, reducing burdens for researchers and respondents (in comparison to mapping of water sources, microbiological testing, or lengthy interviewing) and facilitating improved monitoring and evaluation of water interventions by governments and NGOs. While several studies of household water insecurity have been carried out using qualitative and ethnographic methods (e.g. [11–13]), only a few studies so far have assessed the validity of quantitative measures of household water insecurity [14–17].

The validity of quantitative measures of household water insecurity can be evaluated by comparing their performance in relation to plausible covariates such as microbiological quality of water and psychological stress. *A priori* we would expect that insecure access to water would lead to psychological distress, especially among women, who bear the majority of responsibility for water collection in resource-poor settings [18–23], and that water insecurity would increase with distance from sources and when water is of poorer quality. In accordance with these expectations, studies in Bolivia (using a 4-item scale) and Ethiopia (using a 24-item scale) have shown that household water insecurity measures are associated with anxiety (about health risks for oneself or family members) and shame (when water supply is inadequate for maintaining personal hygiene) [14, 16]. And a study in Uganda (using a 9-item scale) has demonstrated that household water insecurity is lower among those with access to an ‘improved’ source, and higher for those more distant from water sources and those using water sources with higher bacteriological load [17].

One way of assessing the validity of water insecurity measures that has not yet been tried is to assess their sensitivity to changes in water access over time, for example when water access is improved. Nor has any prospective study assessed whether changes to water insecurity are associated with changes in psychological distress. The study reported here employed an intervention-control design to assess household water insecurity and women’s psychological distress before and after improvements to communal water supplies in South Wello (Amhara region), Ethiopia. We employed a water insecurity questionnaire developed through extensive qualitative work in South Gondar (Amhara region), Ethiopia. Previous cross-sectional results from South Gondar indicated that household water insecurity was associated with women’s psychological distress [16]. We were interested in assessing the validity of the water insecurity scale in the context of an intervention, and in the intervention’s implications for water insecurity and psychological distress. Specifically we investigated (1) whether improvements to community water supply reduced water insecurity as measured by our scale, (2) whether the water intervention affected women’s psychological distress, and (3) the impacts of water security on psychological distress, independent of socio-economic status, food security, and harvest quality.

## Methods

### Study setting

This study was conducted in South Wello (Amhara Region) in the highlands of northern Ethiopia, between March 2013 and April 2014. Water access in rural Ethiopia is poor, with an estimated 60% of rural households relying on unimproved sources, such as rivers, ponds, and open springs [2, 24, 25]. Water availability in the highlands is also subject to pronounced seasonal variation, with long rains between May and September (*kiremt*), short rains in February/March (*belg*), and a dry season from October to February (*bega*) [26]. As is common in sub-Saharan Africa, primary responsibility for water collection in rural Ethiopia is usually held by women [27].

Among the households included in this study, approximately two-thirds relied exclusively on smallholder farming for subsistence; the remainder supplemented farming activities by raising animals for sale or weaving cloth. All participants were Muslim and spoke Amharic as a first language.

### Research design

The study had a pre/post test design, with intervention and control sites. In 2013–2014, Catholic Relief Services (CRS), an international non-governmental organization operating in Ethiopia, carried out water improvements in Kalu and Kelela *woredas* (districts) of South Wello, Amhara Region, constructing cement housings (collection boxes) and installing faucets in villages where previously water had been accessible mainly from unprotected springs. This intervention potentially protected water sources from contamination caused by run-off from human or livestock waste, and from contamination that can occur during water collection: At unprotected springs, water vessels are filled by repeatedly dipping a cup into the pool where water seeps up from the ground.

*Sampling*: The sampling unit was the *got*–a cluster of households within the lowest level of the government administrative system, the *kebele*. For ease of explanation, we refer hereafter to *got* as “village” with the caveat that the term may not map exactly onto either locals’ or outsiders’ conceptions of “village.” *Woredas* were not randomly selected, and only the intervention villages studied received water improvements (as opposed to all villages in the *woredas*). Assignment to intervention was purposive: Intervention villages were determined by CRS’s local partners ahead of the study. In selecting control sites we sought villages that were as similar as possible to the intervention sites, *i*.*e*. identical in ethnicity and religion, and similar in size (number of households), altitude, access to schools, clinics, and roads, and portfolio of existing water access at baseline—all of these being considered *a priori* as potentially affecting rates of psychological stress and workload (*e*.*g*. distances walked each day). Based on local knowledge, CRS partners nominated control sites that they believed conformed to these criteria, and the comparability of the control and intervention villages in terms of existing water access was documented through community mapping and through questions in a household survey to determine water source types.

In total, six villages (three intervention sites and three controls) were included in the study. Villages were each at least 15km apart, minimizing the possibility of spillover effects which might arise from households in control sites accessing water sources in intervention sites. The baseline survey was conducted in March-April 2013, and the endline survey, assessing changes in reported water security and psychological distress, was conducted in March-April 2014, which was between one and six months after the installation of improved water supplies. One control village was dropped from the sample following data collection because it did not fulfill a key criterion for a control site (i.e. having no protected source of water) and could not be replaced. Each village contained approximately 50–80 households. Within these villages we attempted to interview all female heads of households or wives of heads of household. At baseline we conducted 247 interviews (149 in intervention villages, and 97 in control villages), and at endline we conducted 223 interviews (183 in intervention villages, and 85 in control villages). Loss to follow-up was substantial: Repeated measures (pre- and post-intervention) were available for only 123 households (74 / 149 [50%] in intervention villages and 49 / 97 [50%] in control villages). The final sample therefore consisted of 347 unique interviews, with 124+100 = 224 households interviewed once and 123 households interviewed twice.

The discrepancy in the overlap between households surveyed in the two rounds was apparently due to a government-led natural resource management (soil and water conservation) campaign that occurs each year between January and April. On account of this campaign–participation in which is mandatory for able-bodied citizens–many members of the communities were not at home during the days we carried out our surveys. In the analyses presented below we focus on the 123 households that provided data at both rounds. Logistic regression suggested no significant differences in socio-economic status, household size, or distance to water sources between households followed up and those lost to follow up (Data not shown).

### Measures

The primary research tool was a structured household survey, administered by trained enumerators, to assess household demographics, assets and economic activities, water security, food security, and informant psychological distress.

#### Water insecurity

Items in the water insecurity scale were assembled by pooling reported experiences of water insecurity derived from free-lists and focus group discussions carried out in South Gondar (Amhara Region, Ethiopia) in 2010 [16]. The scale addressed water access, adequacy, safety, and lifestyle factors (Table 1). It comprised 33 yes/no questions divided into six broad themes: perceived safety and sufficiency of water supply (e.g., ‘In the last 30 days did you or anyone in your household drink water that you thought might not be safe for health?’), obstacles to water access, social tension related to water, opportunity costs of water collection, measures taken to economize on water use, and thirst (e.g., ‘Did you or anyone in your household go to sleep thirsty because there was not enough water?’). ‘Yes’ answers were coded as 1, and ‘no’ answers as 0. (See S1 Appendix, under Supporting Information, for full list of items.)

**Table 1:** Measures of water insecurity employed in this study.

Water security: "reliable access to adequate, safe water for an active lifestyle"	
	Measures
**Access**	Time required to walk from home to source
	Time waiting in line at source
**Adequacy**	*Worry over adequacy of water supply*
	*Reduction in amount of water used for various purposes*
	*Going all day without drinking*
	*Going to bed thirsty*
**Safety**	Type of source used for drinking water (protected vs unprotected)
	*Using an undesirable / dirty source*
	*Perception of having drunk dirty water*
**Lifestyle**	*Opportunity costs of water collection*
	*e*.*g*. *foregoing communal activities in order to collect water*

Note: Items in italics (coded as present / absent) were included in the water insecurity questionnaire (see S1 Appendix, for full list of items in the Water Insecurity questionnaire). Non-italic items (coded as categorical or continuous measures) were collected during structured household interviews.

In our previous study in South Gondar we began with 33 items and refined the scale to 24 items that had response rates >97%. In the current study we adopt as a criterion for retaining an item in the scale that it was responded to by >95% of participants. One section of the scale concerning reasons for not collecting water, answered by very few respondents, was dropped, as were other items that applied to a minority of participants (e.g. ‘reducing the amount of water for growing vegetables’, which is relevant only to those with garden plots). The sum of the 21 remaining items to which respondents said ‘Yes’ was treated as a measure of household water insecurity (possible range 0–21).

Exploratory factor analysis on these 21 items revealed 7 factors with eigenvalues >1.0 at both baseline and endline. In the baseline survey, all 21 items exhibited variance, with observed scores ranging from 0 to 18; at endline, 3 items exhibited no variance and were dropped, and observed scores ranged from 0 to 8. At baseline the first factor had an eigenvalue of 5.9 and explained 27% of the variance; at endline the first factor’s eigenvalue fell to 3.4 and explained 19% of the variance. A scree plot suggested a single dominant factor at baseline, but in the endline data, points of inflection were noticeable for the 2^nd^ and the 4^th^ components. The Cronbach’s alpha values for the 21-item scale were 0.84 at baseline and 0.69 at endline, indicating a very high level of internal consistency at baseline, but a lower level of consistency at endline. A Cronbach’s alpha value of 0.70 or higher is commonly considered to indicate an acceptable level of scale reliability [28].

#### Psychological distress

The measure of psychological distress was the World Health Organization Self-Reporting Questionnaire (SRQ), an inventory of symptoms compiled for cross-cultural psychiatric screening [29], and adapted for Ethiopia [30]. The version recommended by the WHO is an inventory of 20 yes/no questions (the ‘SRQ-20’) including questions on anxiety and depression. Each ‘yes’ response is scored as 1, and scores of 7 or above are commonly considered as a threshold for psychiatric referral [29]. The SRQ has been widely used in Ethiopia [31–34] and internationally (e.g. [35]). The time reference period for questions about psychological distress, as for those about water insecurity, was the past month.

#### Socio-economic status and harvest quality

Socio-economic status (SES) was assessed as a function of self-reported private land ownership (in hectares), livestock ownership (covering 11 types of farm animal including oxen, cattle, equids, goats and poultry), and participation in any non-farm work. Each of the 13 constituent variables was standardized, and a principal components analysis was carried out to reduce the data. The first principal component of the covariance matrix explained approximately 20% of variation in the socio-economic variables at both baseline and endline; these principal component values were retained as summary measures of socio-economic status [36, 37].

In addition to this SES measure, which we assume to be relatively stable from year to year, we also asked about harvest quality in the previous year, which we assume varies stochastically according to weather conditions. Respondents’ ratings of the harvest were elicited on a four-point scale (very bad, bad, good, or very good), and recoded into a binary variable (1, 0, representing good or bad harvest).

#### Food insecurity

The Household Food Insecurity Access Scale (HFIAS) is a 9-item scale that assesses food insecurity prevalence (questions include: ‘Within the last 30 days, did you worry that your household would not have enough food?’ and ‘Was there ever a time when there was no food in your household at all?’). Intensity of food insecurity was assessed by follow-up questions asking whether during the previous 30 days these conditions had been experienced rarely, sometimes, or often (coded as 1, 2, or 3 respectively), yielding a total score ranging 0–27 [38]. A very similar questionnaire has been widely used in Ethiopia and elsewhere [39, 40].

### Data collection

Surveys were translated into Amharic and reverse translated into English to ensure accuracy. Following piloting, interviews were conducted in participants’ homes by interviewers with education to diploma or degree level (8 women and 2 men). Each interview took approximately 45 minutes to complete. Neither data collectors nor respondents were informed at baseline as to whether sites were intervention or control villages. Participants provided verbal consent to participate in the study–written consent was not sought on account of very low levels of literacy in the study population, and the stress that would result from requiring participants to sign a form they could not read. Ethical review and approval of the study was carried out by Emory University’s Institutional Review Board. No specific permissions were required for research in Amhara region as a result of preexisting agreement between local authorities and CRS-Ethiopia that their remit as a civil society organization includes monitoring and evaluation of programme activities.

### Data management

Data were recorded in the field with paper and pencil, and entered into Microsoft Excel (Redmond, WA) by an experienced clerk. After cleaning, data from the two phases of research were merged and composite variables for water insecurity, food insecurity, psychological distress, and socio-economic status were created.

### Data analysis

To assess the impacts of the intervention on water insecurity, food insecurity, and psychological distress, we employed a double-difference approach where the parameter of interest was the interaction between year and intervention status. We modeled the interaction between intervention and year using repeated measures linear regression with fixed effects. We also used repeated-measures linear regression with a random-effects estimator to assess the independent influence of water insecurity, SES, household food insecurity, and the quality of the previous year’s harvest on psychological distress. Analyses were run on a dataset containing households that provided data at both baseline and endline (n = 123 for each round). Significance was assessed at an alpha level of 0.05. Analyses were carried out in Stata version 12 (College Station, TX).

## Results

At baseline, 7 households (9%) in the intervention group were using a protected source as their primary source of drinking water, and no household in the control group was doing so. At endline, 56 households (76%) in the intervention group were using an improved source (i.e. a protected spring where the main source was sealed with concrete and made to flow through a pipe rather than seeping from the ground).

Table 2 shows the mean values of the main variables of interest for the intervention and control groups, at baseline and endline. In the intervention group, water insecurity, food insecurity, and psychological distress declined significantly between baseline and endline. In the control group, psychological distress declined, while water insecurity did not change significantly between baseline and endline. For both the intervention and control groups, harvest quality improved between baseline and endline.

**Table 2:** Summary statistics for household food and water insecurity, socio-economic status, and women’s psychological distress: Baseline and endline measures in intervention and control villages in South Wello, Ethiopia (n = 123).

	Control group (n = 49)		Intervention group (n = 74)	
	mean (SD)	mean (SD)	mean (SD)	mean (SD)
	at baseline	at endline	at baseline	at endline
Water insecurity	2.20 (2.08)	2.31 (2.20)	3.05 (3.79)	1.16 (1.67) **
Food insecurity	4.67 (5.39)	4.31 (5.83)	5.57 (5.54)	3.14 (3.81) **
Psychological distress	7.14 (4.58)	4.86 (4.27)**	6.01 (4.47)	3.72 (3.78) **
Socio-economic status	-0.11 (1.66)	-0.24 (1.63)	0.12 (1.72)	0.31 (1.65)
Harvest quality	0.11 (0.32)	0.93 (0.25)**	0.13 (0.34)	0.77 (0.42) **

Notes:

Household water insecurity is measured on a 21-point scale based on formative work in South Gondar, Ethiopia [16]

Food insecurity is measured by the USDA Household Food Insecurity Access Scale (range 0–27) [38]

Psychological distress is measured on a 20-point scale (SRQ-20) [29]

Socio-economic status (SES) score is derived from principal components analysis of land ownership (hectares), livestock (numbers of sheep, goats, oxen, cows, calves, donkeys, horses, mules, camels), and any off-farm work, according to the procedure proposed by Filmer & Pritchett (2001) [37].

Harvest quality is a binary variable, for each household denoting 0 if the past year’s harvest was said to be insufficient, and 1 if the past year’s harvest was said to be sufficient.

** indicates difference between baseline and endline is significant at p <0.01 (paired t-test)

To investigate patterns of change in responses to individual items in the water insecurity scale, we examined differences in responses to the component questions across the two rounds of the study. Table 3 shows changes in responses to the 21 items in the water insecurity scale between baseline and endline for households in the intervention group and those in the control group. The largest change was in the proportions of women who reported drinking water that they felt was unsafe, which declined significantly in the intervention group but not in the control group. Households in the intervention group also registered substantial declines in reported worry over water, in reducing amounts used for drinking and bathing, and in the practice of borrowing water from neighbors.

**Table 3:** Changes in experiences of household water insecurity between baseline and endline, in intervention and control groups (paired t-tests).

		Intervention (n = 74)	Control (n = 49)
Water Insecurity items (scored 1 / 0)		mean change (95% C.I.)	mean change (95% C.I.)
1	worried about water	-0.15(-0.06, -0.25)**	0.00(-0.13, 0.13)
2	reduced amount … for drinking	-0.14(-0.22, -0.05)**	-0.02(-0.06, 0.02)
3	reduced … cooking	-0.05(-0.15, 0.04)	0.02(-0.09, 0.14)
4	reduced … watering livestock	-0.03(-0.09, 0.03)	0.07(-0.01, 0.15)
5	reduced … washing dishes	-0.04(-0.12, 0.04)	-0.08(-0.16, -0.01)*
6	reduced … bathing	-0.15(-0.24, -0.05)**	-0.04(-0.12, 0.08)
7	reduced … hand washing	-0.03(-0.08, 0.03)	0.04(-0.02, 0.09)
8	reduced … washing before prayer	-0.05(-0.12, 0.01)	0.00 (0.00, 0.00)
9	reduced … washing clothes	-0.06(-0.13, -0.01)*	0.00 (0.00, 0.00)
10	reduced … house-cleaning	-0.09(-0.18, -0.01)*	0.08(-0.02, 0.18)
11	drank water you thought not safe	-0.28(-0.39, -0.17)**	-0.04(-0.22, 0.13)
12	did not cook desirable food	-0.05(-0.11, -0.01)**	0.06(-0.03, 0.15)
13	missed sleep on account of water	-0.09(-0.24, 0.05)	-0.06(-0.26, 0.13)
14	used dirty source	-0.03(-0.10, 0.05)	0.10(-0.06, 0.26)
15	took water from neighbor	-0.18(-0.29, -0.05)**	0.04(-0.13, 0.22)
16	neighbor took water from you	-0.11(-0.24, 0.02)	0.10(-0.06, 0.25)
17	didn't complete work b/c of water	-0.08(-0.17, 0.01)	-0.02(-0.09, 0.13)
18	quarreled with neighbor over water	-0.05(-0.11, -0.01)*	0.00(-0.10, 0.10)
19	went to bed thirsty	-0.05(-0.11, 0.00)*	0.00 (0.00, 0.00)
20	didn't drink all day	-0.03(-0.06, 0.01)	0.00 (0.00, 0.00)
21	missed important events b/c of water	-0.09(-0.22, 0.03)	-0.23(-0.40, -0.06)*

Note: See S1 Appendix for full list of water insecurity questionnaire items.

* indicates difference between baseline and endline is significant at p <0.05 (paired t-test)

** indicates difference between baseline and endline is significant at p <0.01 (paired t-test)

### The impact of improved community water supply on reported water security

In the villages that received the water supply intervention, household water insecurity scores declined by approximately 2 points from baseline to endline, relative to the change in control villages (beta -1.99, 95% Confidence Interval [CI] -3.14, -0.84) (Table 4). Since the standard deviation of the water insecurity scale at baseline was 3.24, this effect corresponds to a downward shift of 0.61 in standard deviations. This effect was robust to control for SES: when SES was added, the coefficient for intervention*time declined only slightly, to -1.88 (95% CI -3.01, -0.76). The effect of the intervention on food insecurity was also substantial (beta -2.07, 95% CI -4.23, 0.09). We did not find evidence for a substantial impact of the intervention on psychological distress (beta -0.12, 95% CI -1.87, 1.84).

**Table 4:** Impacts of water improvement on household water insecurity and women’s psychological distress: Repeated-measures linear regression, with fixed-effects estimator (n = 123).

	Model 1: Outcome = Water insecurity		Model 2: Outcome = Food insecurity		Model 3: Outcome = Psychological distress	
	beta (95% CI)	p	beta (95% CI)	p	beta (95% CI)	p
Year * Intervention	-1.99 (-3.15, -0.84)	<0.01	-2.07 (-4.23, 0.09)	0.06	-0.12 (-1.87, 1.84)	0.99
**Year** (2013 = 0, 2014 = 1)	0.10 (-0.67, 0.88)	0.79	-0.36 (-2.16, 1.42)	0.69	-2.28 (-3.77, -0.80)	<0.01

Note: Intervention was coded 1 for households in villages that received improvements to community water supply (protection of springs) and 0 for households in villages that did not receive such improvements. For definitions of outcome variables, see notes to Table 2.

### Assessing the impact of water insecurity on psychological distress

In repeated measures regression with women’s psychological distress as outcome, household water insecurity remained a significant predictor of psychological distress when household food insecurity, SES, and the quality of the previous year’s harvest were controlled (Table 5). Put differently, each one-point difference on the water insecurity scale was associated with a 0.25-point difference in psychological distress. A change in water insecurity from the 25^th^ to the 75^th^ percentile was associated with a 1-point rise in psychological distress, from 4.7 to 5.7 on the WHO psychological distress scale.

**Table 5:** Impact of selected variables on women’s psychological distress: Repeated-measures regression with random-effect estimator (n = 123).

	Outcome = Psychological distress		
	beta	(95% CI)	p
Water insecurity	0.25	(0.06, 0.42)	0.01
Food insecurity	0.29	(0.18, 0.41)	<0.01
Harvest quality	-1.90	(-2.80, -0.97)	<0.01
Socio-economic status	-0.04	(-0.38, 0.30)	0.80

Note: Model r-square = 0.33. For variable definitions see notes to Table 2.

## Discussion

To our knowledge, this is the first study to assess the impacts of improvements to community water supply on household water insecurity and women’s psychological distress. We found that the intervention was followed by decline in household water insecurity, indicating that our measure of water insecurity is robust to real changes in water access. The difference-in-difference estimate of the intervention effect was equivalent to 2 points on the water insecurity scale, which is more than half of the baseline standard deviation in water insecurity–a substantial effect according to conventional rules of thumb. We did not find evidence of a direct impact of the intervention on women’s psychological distress. This study provides further evidence of construct validity for the water insecurity scale developed from qualitative research in South Gondar [16], and corroborates the findings of other researchers who have investigated relationships between household water insecurity, psychosocial stress, and water quality in cross-sectional studies [14; 15; 17; 41].

Other aspects of the changes we documented in water insecurity, food insecurity, and psychological distress in the course of this study also deserve discussion. In multivariable models in which we assessed the impacts of the intervention, there was evidence of impact not only on water insecurity (p <0.01) but also on food insecurity (p = 0.06) (Table 4). Furthermore, the single strongest predictor of declines in psychological distress was improvements in harvests from 2013 to 2014, presumably due to increased rainfall (Table 5). Household food insecurity was also a significant predictor of psychological distress, and the magnitude of this effect was similar to that of water insecurity.

The impact of the intervention on food security is notable, since it might be taken as a ‘falsification outcome’ in that a clear causal pathway between spring protection and improved household food security is lacking [42]. However, the connections between food and water insecurity are intricate [43] and there may be some common mechanism at work in the outcomes we have described. For example, while the purpose of the intervention was primarily to protect springs from contamination, the fact that the improved springs make it possible to fill jerrycans directly from a faucet (as opposed to dipping water from a pool using a cup) might make water collection faster, and the water thus made available might be used for watering garden plots, and contribute to household food security. We intend to investigate this hypothesis in future.

It is important that we note certain limitations of this study as a test of the relationship between community water improvement, household water insecurity, and psychological distress. First, the gold standard in intervention studies is random assignment, and our study design was limited by purposive assignment (the villages in which CRS had planned water interventions), by incomplete enumeration of the study communities at baseline and endline, and by a relatively small effective sample size (n = 123). Given that our design was observational and the analysis was conducted on the subset of the communities that were available for interview during our study visits, some caution is warranted in interpretation of our results.

Second, the intervention that we focused on did not change the proximity of water supplies to people’s homes, and also left unchanged other likely sources of psychological distress including women’s responsibility for water collection and concerns over rainfall and livelihood security more generally. The relatively small proportion of variance in psychological distress explained by our multivariable model (r-squared = 0.33) indicates the breadth of unmeasured factors (beyond food and water insecurity, harvests, and SES) that are relevant to women’s mental health. We would expect to see yet larger gains in psychological well-being from projects that address more components of water insecurity (e.g. easier access to water sources, improved reliability of supply, or more adequate quantity of water available) and livelihood security more broadly.

Third, the short time interval that elapsed between intervention and follow-up, with endline surveys carried out between one and six months after protection of water sources, constitutes a further limitation to generalization from this study. While a short interval between intervention and follow-up is advantageous in that it minimizes the possibility of influence from undetected forces, it is possible that some impacts of water interventions may take a longer time to be felt. For example, if the relationship between improved water supplies and psychological distress were mediated by declines in child morbidity or mortality, impacts might be felt only after a year or more.

Finally, as demonstrated in exploratory factor analysis, the scale of water insecurity that we used is multidimensional, raising the possibility that different parts of the scale may be responding to different aspects of change in water supply. For example, although water quality might have improved, changes in governance of the newly protected springs might have ramifications for access (e.g. if fees were levied on water users for maintenance of the new infrastructure), and different parts of our 21-point scale may pick up on different aspects of these changes. In South Gondar, where the scale was first developed, we suggested the scale reflected six broad dimensions or themes (including opportunity costs of water collection, social interactions related to water, and perceived safety of water supply), and we advocated its value as a holistic measure of water insecurity in the cultural context of rural highland Ethiopia [16]. While the South Wello setting is similar to South Gondar in language and ethnicity, we found that one section of the water insecurity scale (regarding obstacles to water access) had very low response rates in South Wello relative to our earlier study. The fact that the internal consistency of the scale as measured by Cronbach’s alpha declined in the second wave of data collection–after the intervention, and notably after a season of good rains–may indicate that the scale is better suited to measuring situations of high water stress. The tools developed by Wutich [14, 15] and Tsai and colleagues [17] are to be recommended for researchers who seek unidimensional scales of household water insecurity.

### Conclusion

This study, the first to use a prospective intervention-control design to assess the impact of a water supply intervention on water insecurity and psychological distress, contributes to the evidence of important, under-studied and under-reported health impacts of poor access to water. Our findings emphasize the importance of equitable and universal access to water as articulated in the Sustainable Development Goals. Women are the primary bearers of household duties in Ethiopia and throughout most of sub-Saharan Africa, meaning that they bear the burden of water collection and psychological distress resulting from poor, intermittent, and seasonal access. A shift in focus of water programs from diarrheal disease to more holistic approach to health and well-being is underway, but overdue. Additional research on how to improve water, sanitation, and hygiene programs to accommodate the needs of women is urgently needed.

## Supporting Information

S1 Appendix Water insecurity questionnaire. Full list of items included in the water insecurity questionnaire.
(DOCX)
