# Supplementary material for: Community Water Improvement, Household Water Insecurity, and Women’s Psychological Distress: An Intervention and Control Study in Ethiopia
Source: PLoS One. 2016 Apr 28;11(4):e0153432. doi: 10.1371/journal.pone.0153432 (PMC4849673; doi:10.1371/journal.pone.0153432)
Supplement: S1 Appendix — Full list of items included in the water insecurity questionnaire. (DOCX) [file pone.0153432.s001.docx]

Appendix 1: Water insecurity questionnaire

| 1 | In the past 30 days, did you worry that you would not have enough water for all of your household needs? |
| --- | --- |
| 2 | In the past 30 days, did you reduce the amount of water you used for ____ because there was not enough water or because it was too difficult to collect water? *(Insert items from 2a- 2l in the blank)* |
| 2a | For drinking? |
| 2b | For cooking? |
| 2c | For making *tella* (homebrewed beer) / coffee? |
| 2d | For planting and watering vegetables? |
| 2e | For watering crops? |
| 2f | For watering cattle, sheep and goats? |
| 2g | For washing utensils? |
| 2h | For bathing? |
| 2i | For washing hands? |
| 2j | For washing before prayer? |
| 2k | For washing clothes? |
| 2l | For cleaning the house/ for plastering floor using cow dung? |
| 3 | In the past 30 days, did you or anyone in your household drink water that you thought might not be safe for health? |
| 4 | In the past 30 days, did you or someone in your household not cook a desirable food because there was not enough water? |
| 5 | *[If household contains male school-children]* In the past 30 days, did any boy children in your household, who is a student, go to school late or stay home from school to help with water collection? |
| 6 | *[If household contains female school-children]* In the past 30 days, did any girl children in your household, who is a student, go to school late or stay home from school to help with water collection? |
| 7 | In the past 30 days, did you or anyone else in your household sleep very few hours because they wake up very early in the morning to go for collecting water? |
| 8 | Within the past 30 days, was there any time that you or anyone else in your household did not collect water when you wanted to? [*If no, go to 9; if yes:*] Was it because… |
| 8a | It was too far away/ takes too long to get there? |
| 8b | It was too risky/dangerous for life? |
| 8c | Takes too long to wait at source/queue too long? |
| 8d | Not enough water at the source? |
| 8e | Was too sick or weak to collect water? |
| 9 | In the past 30 days, did you or any member of your household collect water from an undesirable or dirty source because you could not collect from your preferred source? |
| 10 | In the past 30 days, did you or anyone else in your household, because of a lack of water at home, take water from a neighbor? |
| 11 | In the past 30 days, did anyone who is not a member of your family take water from your house because of shortage? |
| 12 | In the past 30 days, were you or anyone in your household unable to complete all of your work due to water collection? |
| 13 | In the past 30 days, have you or anyone else in your household had a quarrel with a neighbor or other person related to collecting water? |
| 14 | *[If married]* In the past 30 days, have you had a quarrel with your spouse about the water needs of your household? |
| 15 | In the past 30 days, did you or anyone else in your household go to sleep thirsty because there was not enough water? |
| 16 | In the past 30 days, did you or anyone else in your household go a whole day without drinking water because there was not enough water? |
| 17 | *[If married]* In the past 30 days, have you had a quarrel with your spouse about not completing daily work? |
| 18 | In the past 30 days, did you or anyone in your household not participate in church / mosque, not call on someone who was ill, or not attend a funeral, wedding, or community meeting when you wanted to because of water collection duties? |
